# Supplementary material for: Thermodynamics, Kinetics, and Mechanisms of the Co-Removal of Arsenate and Arsenite by Sepiolite-Supported Nanoscale Zero-Valent Iron in Aqueous Solution
Source: Int J Environ Res Public Health. 2022 Sep 10;19(18):11401. doi: 10.3390/ijerph191811401 (PMC9517050; doi:10.3390/ijerph191811401)
Supplement: Supplementary file 1 [file ijerph-19-11401-s001.zip › ijerph-1847550-supplementary.pdf]

# Supplementary Materials

**Figure numbers: 5**

**Table numbers: 2**

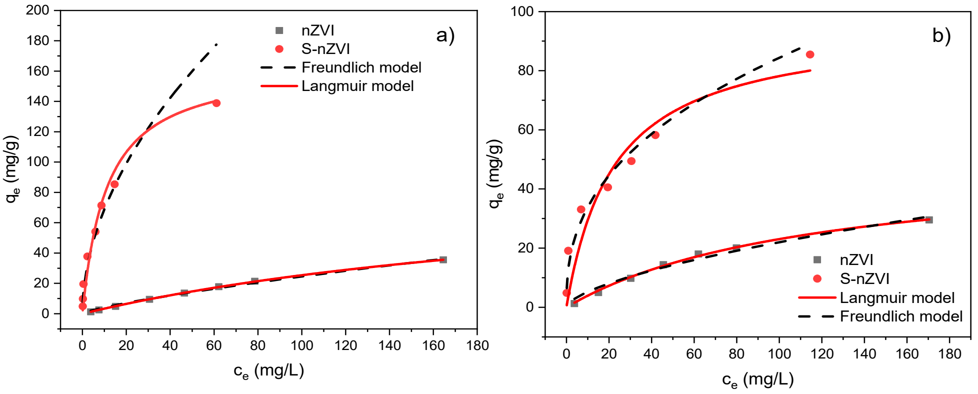


**Figure S1** Adsorption isotherms of a) As(III) and b) As(V) on nZVI and S-nZVI

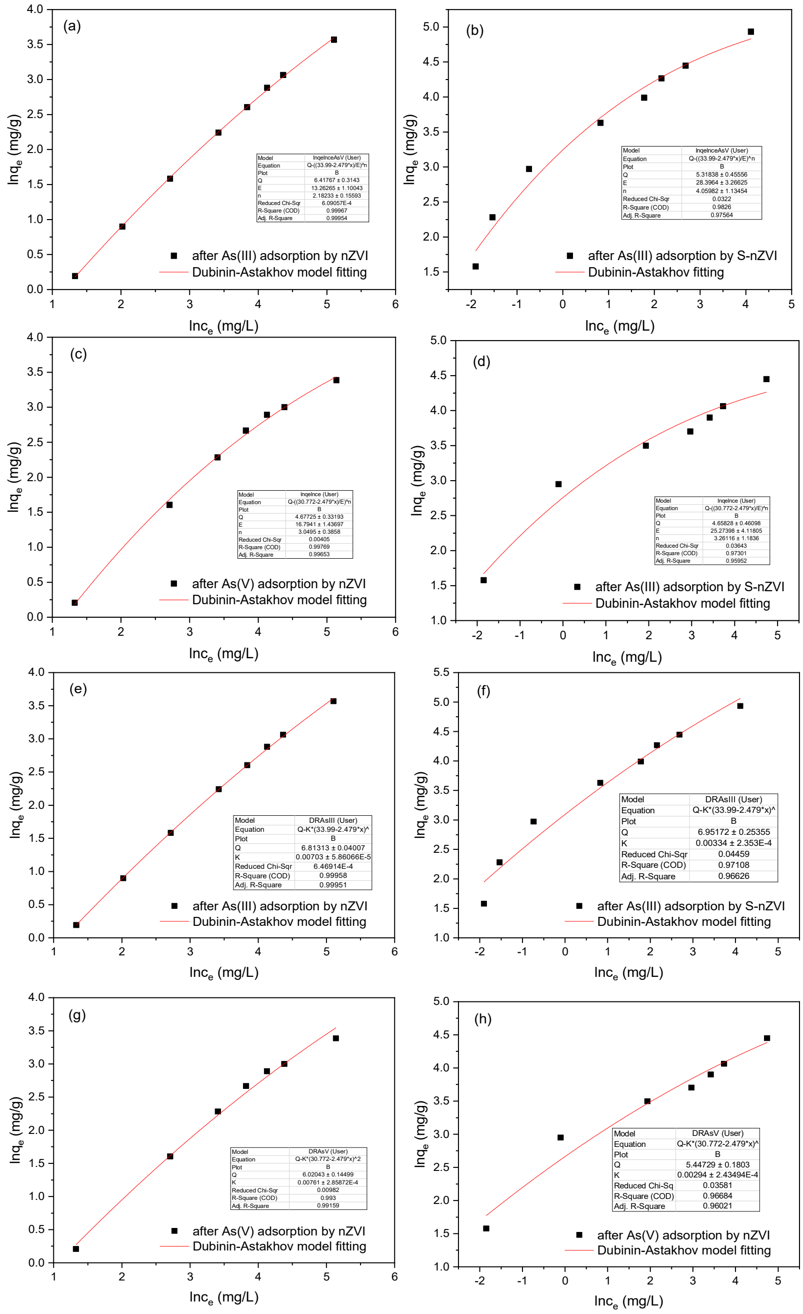


**Figure S2** The Dubinin-Astakhov model fitting results of As(III)/As(V) adsorption isotherms on nZVI and S-nZVI

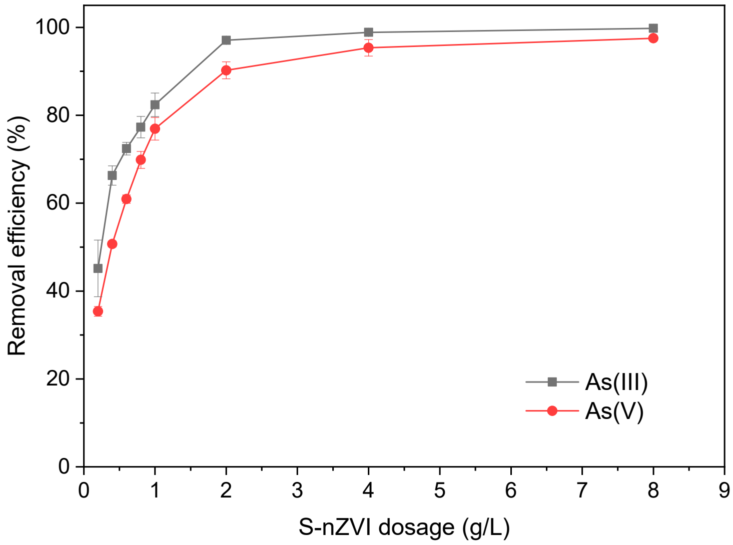


**Figure S3** Effect of S-nZVI dosage on the As(III) and As(V) removal

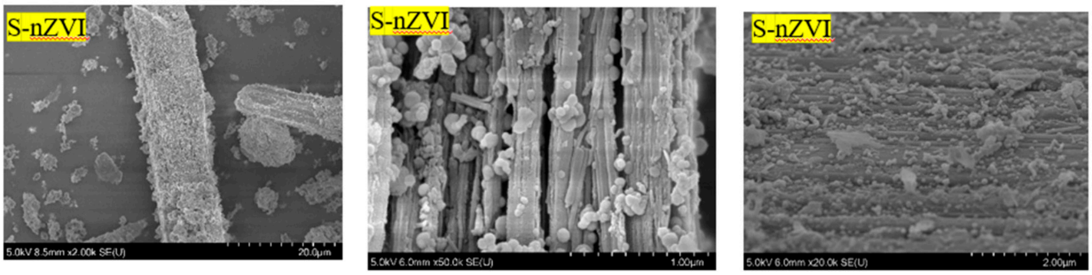


**Figure S4** SEM images of S-nZVI.

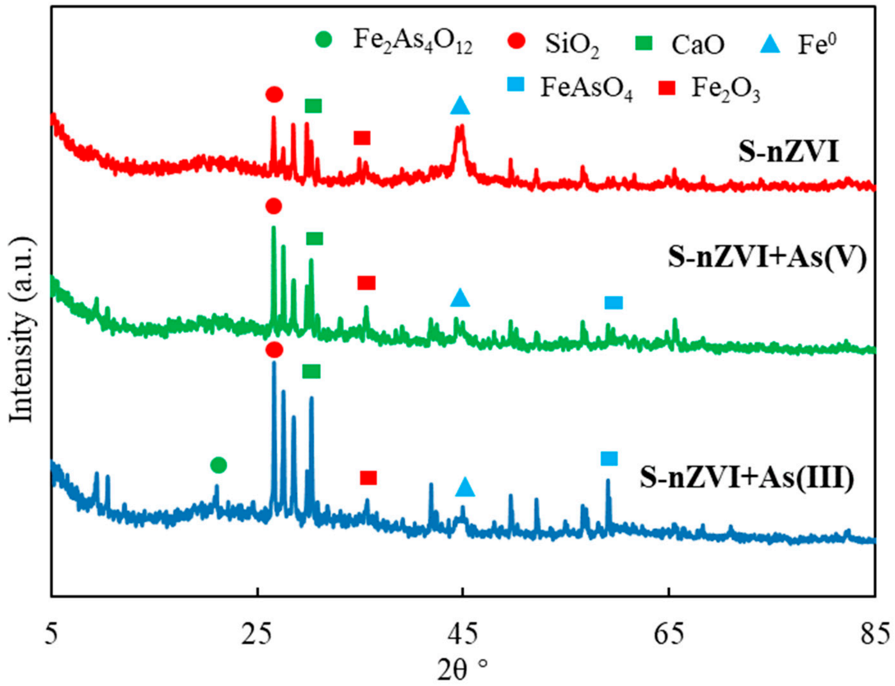


**Figure S5** XRD patters of S-nZVI before and after adsorption of As(III) and As(V)

**Table S1** Comparison of the maximum As(III)/As(V) adsorption capacity by adsorbents reported in literatures.

| Materials | pH | As(III) (mg/g) | As(V) (mg/g) | Dosage (g/L) | Reference |
|---|---|---|---|---|---|
| Zeolite-nZVI | 7.0 | 11.52 | - | 0-30 | (Li et al., 2018) |
| Iron oxides | 5 | 6.77 | 7.23 | 0.5 | (Wang et al., 2013) |
| GB-nZVI | 7.0 | 181.5 | - | 0.1-0.4 | (Liu et al., 2020) |
| Activated char and nZVI | 6.5 | 18.2 | 12.0 | 0.1-3.0 | (Zhu et al., 2009) |
| fuller's earth-nZVI | 7/3 | 50.08 | 91.42 | 1 | (Yadav et al., 2016) |
| Mn-nZVI | 7.0 | 59.90 | 45.50 | 1 | (Bhowmick et al., 2014) |
| S-nZVI | 7.0 | 230.29 | 155.74 | 0.2-8 | This research |

**Table S2** Fitting results of As(III) and As(V) adsorption on nZVI and S-nZVI by Dubinin-Astakhov model (a)-(d) and Dubinin- Radushkevich model.

| | **Dubinin-Astakhov model** | | | | **Dubinin- Radushkevich model** | | |
|---|---|---|---|---|---|---|---|
| | **$lnq_{mD-A}$** | **$E_{D-A}$** | **$n_{D-A}$** | **$R^2$** | **$lnq_{mD-R}$** | **$K_{D-R}$** | **$R^2$** |
| | | | | As(III) | | | |
| nZVI | 6.42 | 13.26 | 2.18 | 0.999 | 6.81 | 0.007 | 0.999 |
| S-nZVI | 5.32 | 28.4 | 4.06 | 0.982 | 6.95 | 0.003 | 0.971 |
| | | | | As(V) | | | |
| nZVI | 4.68 | 16.79 | 3.05 | 0.998 | 6.02 | 0.008 | 0.993 |
| S-nZVI | 4.66 | 25.27 | 3.26 | 0.973 | 5.45 | 0.003 | 0.967 |
